# Early morning hour and evening usage habits increase misinformation-spread

**DOI:** 10.1038/s41598-024-69447-8

**Published:** 2024-08-30

**Authors:** Elisabeth Stockinger, Riccardo Gallotti, Carina I. Hausladen

**Affiliations:** 1 Computational Social Science, Department of Humanities, Social and Political Sciences, ETH Zurich, Zurich, 8092 Switzerland; 2 Complex Human Behaviour Lab, Fondazione Bruno Kessler, Trento, 38123 Italy

**Keywords:** Human behaviour, Misinformation spread, Diurnal patterns, Social media, Computational social science, Circadian rhythms and sleep, Computational science

## Abstract

Social media manipulation poses a significant threat to cognitive autonomy and unbiased opinion formation. Prior literature explored the relationship between online activity and emotional state, cognitive resources, sunlight and weather. However, a limited understanding exists regarding the role of time of day in content spread and the impact of user activity patterns on susceptibility to mis- and disinformation. This work uncovers a strong correlation between user activity time patterns and the tendency to spread potentially disinformative content. Through quantitative analysis of Twitter (now X) data, we examine how user activity throughout the day aligns with diurnal behavioural archetypes. Evening types exhibit a significantly higher inclination towards spreading potentially disinformative content, which is more likely at night-time. This knowledge can become crucial for developing targeted interventions and strategies that mitigate misinformation spread by addressing vulnerable periods and user groups more susceptible to manipulation.

## Introduction

Collective intelligence and democracy rest on the shoulders of public free access to unbiased and diverse information^1,2^. Social media blurs the borders between news creation, consumption, and distribution^3^, as well as between personal communication, announcements from individuals, fiction, and advertisement. Along with the optimization criteria employed in recommendation algorithms^4,5^ and network structures, this contributes to the creation and spread of mis- and disinformation online^3^, to political manipulation^6–10^, a collapse of content diversity^11–13^ and political polarisation^14^.

This leaves the responsibility to distinguish between the content types and discern truth from deception to the user. However, our ability to scrutinise new information for its reliability depends on the individual’s internal state. Cognitive resources and one’s thinking style^15–27,27–31^, as well as emotional state^19,32–35^, have been explored extensively in this regard with diverging results. Other influential factors include cognitive biases and prior beliefs^3,27,36–40^.

These factors are not constant but exhibit regular cyclical behaviours with periods ranging from hours to seasons^41–45^ and depend on external factors such as light exposure^46–48^, atmospheric conditions^49,50^, social interactions^48^, or the device used to access social media^51–54^. These external or environmental factors act as *zeitgebers*, entraining or synchronising the human biological rhythm. Inter-individual differences affect circadian process timings as well. A process is referred to as circadian if it recurs endogenously on a twenty-four-hour cycle, and as diurnal if there is a recurrence which may or may not be endogenous. These inter-individual differences include diverging phase preferences known as chronotypes^55^. In the absence of disruptions to one’s natural rhythms, chronotypes perform better at their optimal times with “evening types” (or “night owls”) achieving better results in the evening, and “morning types” (or “early birds”) in the morning^56^. Depending on environmental or social constraints, sleep and activity timings may be out of phase with one’s internal circadian time, leading to deterioration in cognitive performance such as attention, memory, or decision-making capacity^56^ as well as reflective thinking^57^. Finally, sleep loss itself has long-reaching effects such as reductions in altruistic behaviour^58^.

In an additional layer of complexity, social media are dynamic: They follow human circadian or diurnal rhythms,^59,60^ or the weekday-weekend rhythms^41,61^. The timing of a Twitter (now X) post is an essential factor in its spread and popularity^45^. Clock time and sunrise/sunset hours have distinct impact on tweeting activity^41^.

Despite all efforts to mitigate mis- and disinformation^62–65^, the problem is even rising in importance with geopolitical ^e.g.^^66^ and epidemiological developments ^e.g.^^22^. Especially the global COVID-19 pandemic has invited a new wave of conspiracy theories^22^, with up to a third of the population believing COVID-19 to have been bio-engineered^22^. As an event with drastic and synchronous impact across a major part of the population, the pandemic may have contributed fundamentally to polarisation^67^.

These developments may have cascading effects: higher exposure to COVID-19 misinformation has been linked to increased mistrust in information and to lower confidence in judging its veracity^68^. Similarly, the use of social media for news consumption can increase the accuracy one attributes to misinformation^69^. These effects may lead to a feedback loop eroding the users’ ability to critically judge new information.

**Figure 1:**
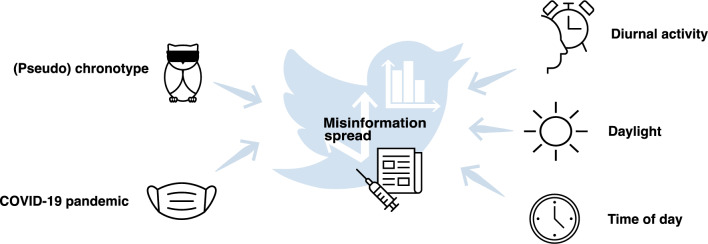
Factors influencing the spread of mis- and disinformation, containing daylight, time of day, human diurnal activity, (pseudo) chronotype, and the COVID-19 pandemic. We use the term (pseudo) chronotype to refer to user archetypes based on diurnal tweeting activity.

We contribute to this literature by investigating mis- and disinformation on social media^70^ with an analysis of the interaction effects between temporal rhythms of disinformative content and social media usage in the context of COVID-19. Specifically, we aim to answer the research question of how the spread of mis- and disinformation on Twitter varies throughout the day. We use the terms “Twitter” and “Tweet” in this paper as our data was collected before the rebranding into X. Additionally, we explore whether there are individual differences in users’ propensity to spread mis- and disinformation on Twitter based on their typical diurnal activity patterns, both during the day and as a general inclination. Figure 1 visualises these connections.

## Results

We analysed a secondary Twitter dataset^71^ relating to the COVID-19 pandemic. Only tweets containing a link to another website were included in the dataset and classified into nine categories, also called content types, according to an expert rating of the reliability of the link’s domain (see “Diurnal cluster activity” for details). Content that is *politically biased* (aiming to build a consensus on a polarised position by omission, manipulation and distortion of information), *fake or hoax* (entirely fabricated or manipulated content that aims to be perceived as realistic and reliable) or *conspiracy or junk science* (strongly ideological, inflammatory content alternative or oppositional to tested and accountable knowledge and information, with the intent of building echo chambers) may have been (but need not be) designed with the purpose of manipulation or affectation. We therefore consider these content types to be “potentially disinformative”. This group stands against the other six categories of *Science*, *Mainstream media*, *Satire*, *Clickbait*, *Other*, and *Shadow*. While Satire and Clickbait are not dependable sources of information, they usually are easily identified and are not likely to have the intention of manipulating opinions. The category *Other* collects content which is not easily classifiable, while *Shadow* includes anonymized links that were not possible to expand. We merged *Other* and *Shadow* in this paper, the reliability of both is unknown. The categories alongside their user activity statistics are described in Supplementary Table S1.

### Four archetypical activity patterns

Our analysis focuses on the individual usage patterns on Twitter and their daily fluctuations. To that end, we first compute the average posting activity of each user over the day, including Tweets, Retweets, and Replies. We then use k-means clustering to group the average posting activity curves. The analysis reveals the presence of three distinct clusters with unique patterns of posting activity. Users with low post rates ($<240$ posts across the time span under analysis) are separated into a fourth cluster. While this paper focuses on Tweets originating from Italy, we conducted the same analysis for Tweets originating from Germany and found these prototypical activity patterns to hold across the two countries (Supplementary Note A).

**Figure 2:**
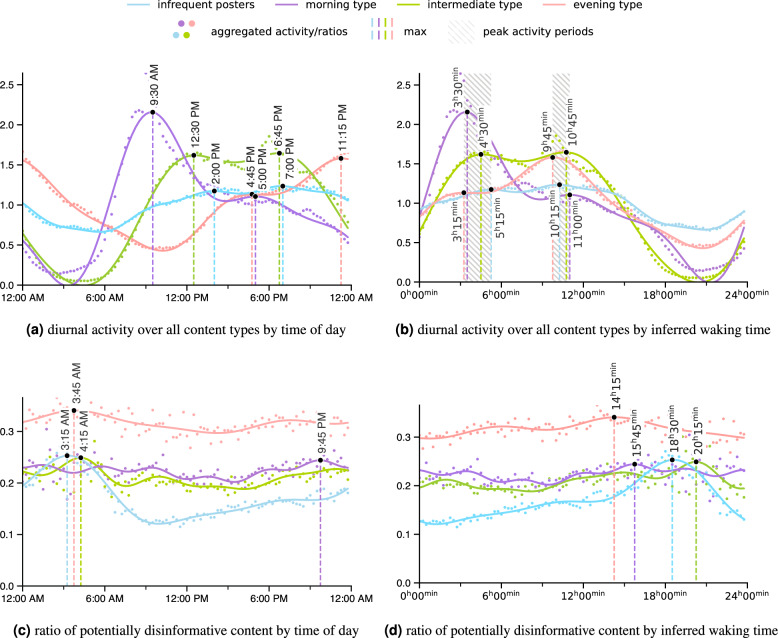
Smoothed diurnal activity ((**a**) and (**b**), see “Diurnal cluster activity”) as well as the ratio of potentially disinformative content posted per cluster ((**c**) and (**d**), see “Content type ratios”). For each cluster, the one (or two) highest peaks of activity and ratio are annotated with their time of occurrence. The shaded area in panel (**b**) stresses the closeness of peak activity after inferred awakening across the clusters.

Figure 2a illustrates the activity patterns of the four clusters throughout the day. Each dot shows how much of the cluster’s posting activity occurs during the given time interval. The curves indicate the smoothed posting activity for each cluster over the day, where the two largest peaks are annotated (given in detail in Supplementary Table S2). We refer to the clusters as morning, evening, and intermediate type posters, named after their respective peak activity times, as well as infrequent type posters (Fig. 2a). While the chosen cluster names are commonly used to refer to chronotypes, we here use them figuratively and without a claim to reflect underlying traits.

Generally, user activity follows a bimodal distribution (Supplementary Table S3 shows the Dip-test results rejecting single-modality). The purple curve represents *morning types*, with the curve reaching its maximum in the morning at 9:30 am at around twice the average value. In contrast, *evening types*, displayed in red, exhibit their highest activity at around 11:15 pm. *Intermediate types*, represented by the green curve, feature two nearly identical peaks in size, with the highest peak occurring around noon. The *infrequent posters* group, represented by the blue curve, shows consistent activity levels throughout the day. This cluster groups users who have contributed only a few posts to the dataset, irrespective of activity distribution throughout the day. As a result, the cluster likely includes users with heterogeneous tweeting behaviours. Their activity patterns may average out over the course of the day, resulting in a relatively flat curve.

We extrapolate from the users’ diurnal activity patterns on Twitter to sleeping and waking cycles, which have previously been linked in literature ^e.g.^^41,42^. These cycles can vary significantly between clusters. We consider the 16 continuous hours of highest aggregated activity a coarse proxy for user’s average waking time. Consequently, we consider activity outside of this interval to represent prolonged wakefulness, where the user is active despite it being a time of habitual rest. A formal definition is given in Eq. (11). Onset and end values of increased activity for each cluster are listed in Supplementary Table S3 (“heightened activity”).

Figure 2b aligns the clusters’ activity by inferred waking time. From this perspective, the diurnal activity curves for each cluster show remarkable similarities. The peaks for all clusters fall within a distinct time window (shaded in grey in the figure). The first peak of activity occurs within 3 h 15 min and 5 h 15 min after inferred awakening within a window of 2 h. The second peak occurs within a window of 1 h 15 min starting at 9 h 45 min after inferred awakening. The sizes of the peaks in activity seem to be as much of a differentiating characteristic for each cluster as the time of occurrence of peak activity. The activity valleys across clusters are similarly close, occurring around 3 h before inferred awakening (Supplementary Table S2).

### *Evening types* spread most potentially disinformative content, *infrequent posters* the least

The clusters show distinct features beyond their typical activity patterns. In particular, we find a significant association between potentially disinformative content type and cluster affiliation ($\chi ^2=28,860.01$, $p$-value$< 0.001$).

Figure 2c shows the diurnal fluctuation of the ratio of potentially disinformative content. Each dot indicates how much of all content with known reliability ratings published within the given time interval was potentially disinformative. The curves represent the smoothed trends of potentially disinformative content ratios (see Content type ratios) throughout the day. Notably, ratios for *evening types*, ranging between 0.27 and 0.37, are consistently higher than for the other clusters (see Table 1 for statistical significance and Supplementary Table S1 for the distinct variation in ratios of content types spread by cluster). *Infrequent posters* exhibit the lowest ratios of potentially disinformative content overall (Table 1). This can again be explained by the definition of this cluster as grouping users with few posts in the dataset, as there is a positive correlation between the amount of posts per user in the dataset and the ratio of potentially disinformative content across all users ($\rho =0.200$, $p$-value$<0.001$) as well as within each cluster (Table 2a).

**Table 1:** One-side d Mann-Whitney U test indicating whether the distribution of ratios of potentially disinformative content throughout the day (see Fig. 2c or 2d) underlying one cluster (rows) is smaller than that of another cluster (columns).

		Morning		Intermediate		Evening	
		*U*	$p$-value	*U*	$p$-value	*U*	$p$-value
Coarse	Infrequent	1419	**6.1e−17**	1818	**2.2e−13**	9	**3.5e−33**
	Morning	–	–	6499	1.0e+00	25	**5.7e−33**
	Intermediate	–	–	–	–	51	**1.3e−32**
Smooth	Infrequent	1507	**4.0e−16**	1873	**6.1e−13**	0	**2.6e−33**
	Morning	–	–	6875	1.0e+00	0	**2.6e−33**
	Intermediate	–	–	–	–	0	**2.6e−33**

Significant values (*p*-value < 0.05) are in [bold].

**Table 2:** Correlation tables in between diurnal and total posting activity and potentially disinformative content activity.

(a) Spearman’s rank correlation coefficient and corresponding $p$-value correlating a user’s total number of posts with ratios of potentially disinformative content									
	Spearman’s $\rho$	$p$-value							
Infrequent	0.162	** 1.2e−02**							
Morning	0.179	** 5.0e−09**							
Intermediate	0.125	** 2.8e−06**							
Evening	0.134	** 1.4e−05**							
Total	0.200	** 2.3e−22**							

(b) Spearman’s rank correlation coefficient and corresponding $p$-value correlating a user’s (a) aggregated activity level without Fourier smoothing (Equation 2, “coarse”) and (b) the smoothed set of diurnal user activity (see “Diurnal cluster activity”, “smooth”) at different time points in a day with the averaged user ratios of politically biased information, fake or hoax news, and conspiracy or junk science as well as all potentially disinformative content (Eq. 13). In row “smooth”, the smoothed set of potentially disinformative content (see “Content type ratios”) was used to compute the correlation coefficient									
		Potentially disinformative		Politically biased		Fake or hoax		Conspiracy & junk science	
		Spearman’s $\rho$	$p$-value	Spearman’s $\rho$	$p$-value	Spearman’s $\rho$	$p$-value	Spearman’s $\rho$	$p$-value
coarse	Infrequent	− 0.324	** 1.3e−03**	− 0.303	** 2.7e−03**	− 0.534	** 2.2e−08**	0.524	** 4.2e-08**
	Morning	− 0.272	** 7.4e−03**	− 0.326	** 1.2e−03**	− 0.105	3.1e−01	0.097	3.5e−01
	Intermediate	− 0.446	** 5.1e−06**	− 0.713	** 3.7e−16**	− 0.081	4.3e−01	0.012	9.1e−01
	Evening	0.135	1.9e−01	0.302	** 2.8e−03**	-0.019	8.6e−01	− 0.194	5.9e−02
	Total	− 0.369	** 2.1e−04**	− 0.398	** 5.9e−05**	− 0.459	** 2.6e−06**	0.614	** 3.0e−11**
smooth	Infrequent	− 0.402	** 5.0e−05**	− 0.321	**1.4e−03**	− 0.593	** 1.9e−10**	0.512	** 9.5e−08**
	Morning	− 0.432	** 1.1e−05**	− 0.308	** 2.2e−03**	− 0.104	3.1e−01	0.097	3.4e−01
	Intermediate	− 0.646	** 1.2e−12**	− 0.670	** 8.5e−14**	− 0.091	3.8e−01	0.064	5.4e−01
	Evening	0.261	** 1.0e−02**	0.306	** 2.4e−03**	0.008	9.4e−01	− 0.197	5.4e−02
	Total	− 0.441	** 7.0e−06**	− 0.410	** 3.3e−05**	− 0.501	** 2.0e−07**	0.611	** 3.8e−11**

Significant values (*p*-value < 0.05) are in [bold].

### Potentially disinformative content spreads at night

While the total number of posts per user is positively correlated with an increased ratio of potentially disinformative content, heightened activity at a given time of day is negatively correlated with spreading potentially disinformative content at that time ($\rho = - 0.369$, $p$-value$<0.001$, Table 2b). This correlation is significant for all clusters except for evening types, and significant for all clusters when considering smoothed content type ratios only.

**Figure 3:**
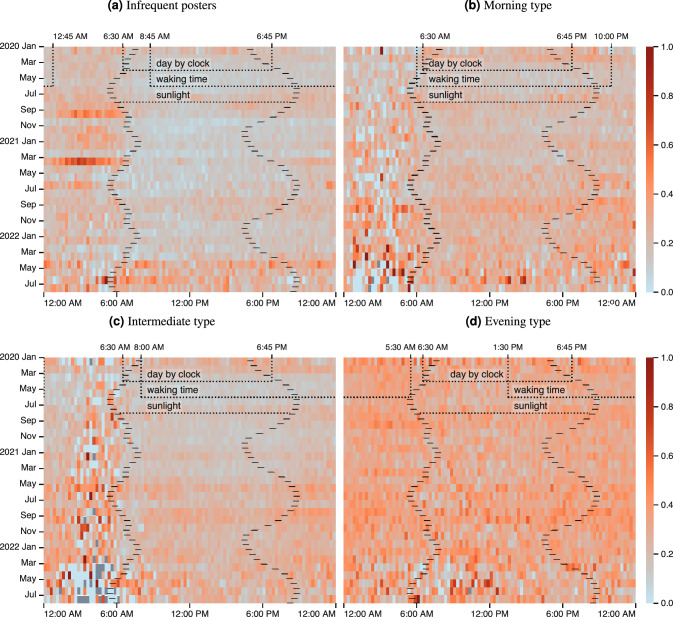
The ratio of potentially disinformative content over time of day on the x-axis, and year and month on the y-axis. The darker red a square, the higher the ratio of potentially disinformative content. The hatched curves indicate the average sunrise and sunset times within a given month. The dashed lines represent the active times per cluster, and the times of day as defined by the clock. Missing values are presented in grey.

One’s tendency to spread potentially disinformative content shows temporal patterns beyond correlations with activity across the day. We analyse three distinct time periods: daytime and nighttime as defined by the clock, by the presence of daylight, as well as by inferred time of regular waking. Figure 3 visually represents these day and night periods for each cluster.

We consider a *day by clock* to occur between 6:30 am and 6:45 pm, the averages of sunrise and sunset throughout the year rounded to the closest quarter hour. These times are marked by connected dashed vertical lines. Many people’s routines and schedules are defined by clock time and therefore consistent throughout the year. *Daylight*, the time period between sunrise and sunset, each represented by hatched curves, varies across the year and across geographic locations. We calculate these times at a monthly granularity at the average locations of the users in our dataset within Italy (sunset and sunrise times differ by less than an hour between any points on the map). Sunlight impacts many physiological and cognitive processes^46,48^, synchronising the human biological rhythm across the population group. *Inferred waking time*, also indicated by dashed vertical lines, is defined per cluster and represents the 16 continuous hours of highest aggregated activity. Activity outside regular waking hours may represent times of impaired cognitive capacity ^e.g.^^72^. In our statistical analysis, we compare the time periods “within” these borders with those “outside” them.

We find particularly strong and regular distinctions between daytime and nighttime activity levels with respect to the spreading of potentially disinformative content and the congruent content types (Table 3).

There is a statistically significant increase in the proportion of potentially disinformative content shared between 6:45 pm and 6:30 am as well as outside daylight hours for all clusters except for morning types ($p$-value$<0.001$ for other clusters). During prolonged wakefulness, only *infrequent posters* publish a significantly higher share of potentially disinformative content ($p$-value$<0.001$). By contrast, the other clusters exhibit a significant reduction in potentially disinformative content spreading in this time frame ($p$-value$=0.039$ for *morning types* and $<0.001$, for *intermediate* and *evening types*).

**Table 3:** Mann-Whitney U test comparing the distributions of content type ratios (see Eqs. 13 and 15) during different definitions of daytime: the day by clock, a day as the time between sunrise and sunset, as well as inferred waking time. We account for a safety margin of $s=1$ h before and after each border value.

		6:30 am–6:45 pm$^{1}$			sunrise–sunset$^{2}$			waking–bedtime$^{3}$		
		*U*	$p$-value	Less	*U*	$p$-value	Less	*U*	$p$-value	Less
Potentially disinformative	Infrequent	434,354	** 5.6e−67**	Day	463,293	** 7.1e−55**	Day	440,930	** 7.7e−27**	Day
	Morning	698,385	1.1e−01	–	701,103	1.5e−01	–	629,323	** 3.9e−02**	Night
	Intermediate	647,216	** 8.1e−06**	Day	657,414	** 9.3e−05**	Day	646,818	** 1.7e−04**	Night
	Evening	604,870	** 1.6e−13**	Day	605,991	** 2.7e−13**	Day	696,449	** 5.3e−09**	Night
Political	Infrequent	431,886	** 4.5e−68**	Day	450,722	** 5.8e−60**	Day	451,253	** 7.4e−24**	Day
	Morning	709,811	3.4e−01	–	712,093	4.2e−01	–	615,595	3.8e−01	–
	Intermediate	696,713	1.6e−01	–	696,426	1.5e−01	–	647,006	** 1.5e−04**	night
	Evening	572,944	** 3.0e−20**	Day	593,292	** 9.0e−16**	Day	758,212	** 2.0e−22**	Night
Fake or hoax	Infrequent	683,876	** 3.3e−03**	Day	678,212	** 1.2e−03**	Day	546,086	** 3.9e−05**	Day
	Morning	872,521	** 2.9e−18**	Night	874,363	** 1.1e−18**	Night	748,896	** 9.8e−22**	Night
	Intermediate	829,162	** 6.7e−11**	Night	844,240	** 1.7e−13**	Night	830,793	** 2.5e−55**	Night
	Evening	642,890	** 2.0e−07**	Day	648,988	** 1.2e−06**	Day	699,848	** 1.4e−09**	Night
Conspiracy & junk science	Infrequent	791,785	** 1.7e−04**	night	781,833	** 1.3e−03**	Night	770,504	** 1.1e−25**	Night
	Morning	814,498	** 1.1e−07**	Night	798,242	** 1.1e−05**	Night	762,932	** 1.7e−25**	Night
	Intermediate	770,202	** 1.7e−03**	Night	770,883	** 1.6e−03**	Night	798,392	** 1.3e−41**	Night
	Evening	776,472	** 3.2e−03**	Night	745,406	3.6e−01	–	552,002	** 2.2e−04**	Day

The $p$-values shown are for one-tailed Mann-Whitney U tests of the distributions of content type ratios during day and night, if significantly different from one another as indicated in the columns. The smaller distribution is indicated in column “Less”. If there is no significant difference between distributions, the $p$-value of two-tailed Mann-Whitney U test is given.  Significant values ( $p$-value $< 0.05$) are in [bold].

$^1$ compares the distribution of ratios *r*(*t*, *c*, *f*) for $t \in [\text {7:30 am} - \text {5:45 pm})$ (“day”) with those for $t \in [\text {7:45 pm} - \text {5:30 am})$ (“night”), considering the safety margin.

$^2$ compares the distribution of ratios between sunrise and sunset (“day”) with those between sunset and sunrise (“night”). The sunrise and sunset times are calculated geometrically using Python’s suntime library https://github.com/SatAgro/suntime for the first day of each month. The locations are calculated at the average location of posts per user and time period in our dataset on the granularity of provinces and cities (territorial units of level 3 as defined by Eurostat^73^).

$^3$ compares the distributions of ratios within $[i(g(c,n),s), i(g(c,n),n-s))$ (“day”) with those of the interval $[i(g(c,n),n+s), i(g(c,n),-s))$ (“night”) for $n=16$. *i*(*t*, *n*) and *g*(*c*, *n*) are defined in Eqs. (9) and (11), respectively.

### Rhythms of potentially disinformative content

The ratio of potentially disinformative content for *morning types* is highest in the late evening at 9:45 pm. For the other clusters, peak times fall in the early morning between 3:15 am and 4:15 am (Fig. 2c). When aligned by inferred waking time (Fig. 2d) the peak times of potentially disinformative content are spread more evenly and across a wider time span, occurring between 14 h 15 min and 20 h 15 min after inferred awakening (Supplementary Table S2).

The amount of data available differs significantly between clusters and times of the day. Especially *morning* and *intermediate* types do not post much in the early morning hours in general, resulting in large variance between consecutive points (see Supplementary Fig. S2). While the peaks of potentially disinformative content fall into time frames of generally low variance for *morning*, *evening* and *infrequent type* users, the peak for *intermediate type* may be caused by low amounts of data.

The peak of potentially disinformative content ratios in the early morning for *infrequent posters* (consisting of users with few posts in the dataset, regardless of their activity rhythms) may be explained on the user level, with users of different activity habits predominating the cluster’s expression at different times. In particular, *evening type* users generally show higher ratios of potentially disinformative content and post more in the early morning. Users whose behavior is akin to *evening types* but who were assigned to the cluster of *infrequent posters* may be responsible for most posts within the cluster in the early morning.

When considering the peaks of potentially disinformative content ratios for *morning* and *evening* type users, we find highest potentially disinformative content ratios at 15 h 45 min and 14 h 15 min after inferred awakening, towards the end of regular waking times. The distance of curves of potentially disinformative content ratios decrease across several metrics when aligning the curves of potentially disinformative content ratios by waking time as opposed to time of day, but increase in others (Supplementary Table S4a).

Content ratios only point to the relationship between potentially disinformative and overall content, not to the behaviour of users spreading potentially disinformative content itself. Therefore, the prevalence of potentially disinformative content during the night hours may be explained by a decreased presence of reliable content, for example due to the reduction of posts by news outlets. Supplementary Fig. S1 shows the potentially disinformative activity curves throughout the day. Qualitatively, these curves and their peak and trough times are similar to those of overall activity (Fig. 2a and b).

### Content type preference is linked to archetypical diurnal tweeting behaviour

We have so far analysed the binary categories of content that is potentially disinformative, and content that is unlikely to be so. There are, however, also interesting observations within the individual content types.

**Figure 4:**
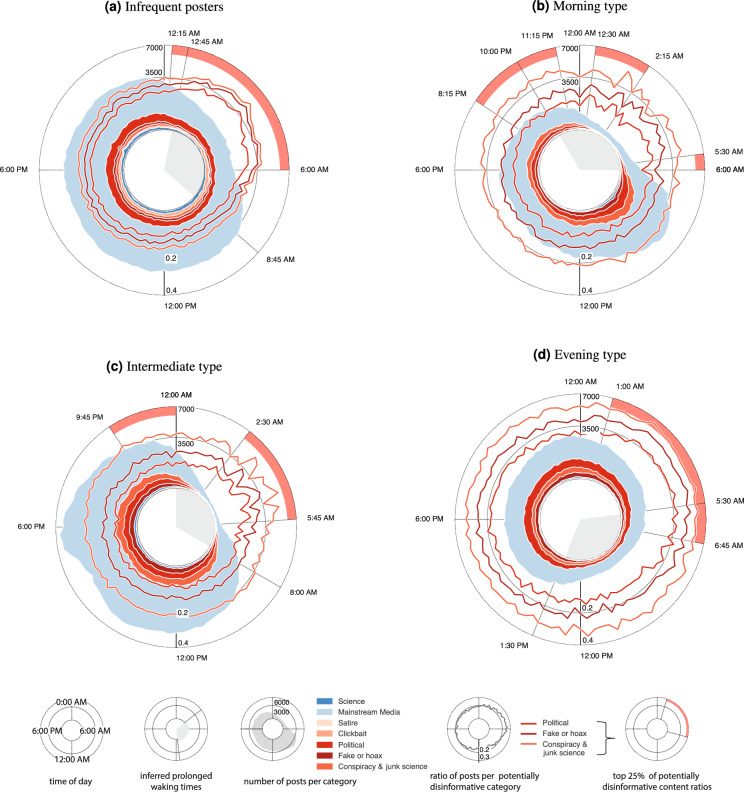
Each panel displays per cluster: the cumulative number of posts with known reliability classification throughout the day (coloured areas), the cumulative ratios of potentially disinformative content types (red lines), the user’s 8 least active hours (inferred prolonged wakefulness, grey inner arc), and the times with the highest quartile of potentially disinformative posts (red outer arcs). The axis scales are shared between panels.

The coloured areas of Fig. 4 represent the activity of all user clusters and individual content types around a 24-hour clock. *Morning* and *evening types* show a particular tendency towards conspiracy theories and junk science, especially as compared to *infrequent types*, who show the strongest inclination towards scientific content of all clusters. Only *intermediate types* spread even more conspiracy and junk science than politically biased content (Supplementary Table S1). However, mainstream media reassuringly make up the vast majority of content spread by all clusters.

The red lines in Fig. 4 represent the cumulative ratios of potentially disinformative content types. Notably, the ratio of conspiracy and junk science increases noticeably during the nighttime when ratios of fake or hoax content and of politically biased content are lowered. The positive correlation of conspiracy theories and junk science with activity throughout the day is, however, only significant for *infrequent posters* ($\rho = 0.524$, $p$-value$>0.001$, Table 2b). This relationship is reversed for *evening type* users, who show a significant positive correlation between activity and politically biased content ($\rho = -0.398$, $p$-value$>0.001$).

Figure 4 also shows the times where one’s tendency to spread potentially disinformative content is in the top quartile ($Q_3$ in a 4-quantile) as red arcs along the graph’s edges. The inner grey arcs represent the time of prolonged wakefulness for each cluster (see also Supplementary Table S3). *Infrequent posters* experience the onset of increased spreading of potentially disinformative content at 12:15 pm, close to their inferred bedtime at 12:45 am and only shortly before *evening type* individuals. *Evening types*, however, only enter prolonged wakefulness at 5:30 am. For *morning* and *intermediate types*, the times of increased tendency to spread potentially disinformative content is split across the day, partly within and partly outside of inferred prolonged wakefulness. For morning types, part of this quartile of increased spreading of potentially disinformative content falls between 8:15 pm and 11:15 pm, earlier than any other cluster. Intermediate type users show an increase from 9:45 pm to midnight and from 2:30 am to 5:45 am.

### The impact of the lockdown

As our dataset collects content related to the COVID-19 pandemic, we must consider the impact of non-pharmaceutical interventions, such as home office or curfews, on daily rhythms, as well as potential changes in the macroscopic informational landscape of Twitter^74^. We specifically consider the time period of Italy’s first lockdown from March 9^th^ to May 18^th^, 2020. The lockdown lead to significant changes in posting activity (potentially disinformative post counts are from different populations, $\chi ^2=1343.13$, $p$-value$<.001$). From the entire span covered by the dataset to this time, all clusters except for *intermediate type* users tweeted more potentially disinformative posts per day and user during the lockdown (e.g. 72.4 % for *evening types*, Table 4). The increase of overall posting activity is even higher (74.9% for evening types). In other words, while users tweeted more during the lockdown, the relative increase in potentially disinformative posts was lower than other types of content (− 6.7% for *evening types*). The reduction of potentially disinformative content ratios during lockdown can likely be attributed to an increase in other content types, likely including a surge of informational coverage driven by mainstream and state media^71^.

**Table 4:** This table shows the percentage of change from the time outside of the first lockdown period in Italy to the lockdown period for overall and potentially disinformative posts per day and user as well as the average ratio of potentially disinformative content posted by users in a cluster (Eq. 15).

	Infrequent	Morning	Intermediate	Evening
Posts per day and user	148.1%	94.7%	50.4%	74.9%
potentially disinformative posts per day and user	110.4%	45.7%	− 2.3%	72.4%
average ratio of potentially disinformative posts per user in cluster	− 16.5%	− 20.2%	− 26.8%	− 6.7%

## Discussion

Propaganda campaigns and targeted manipulation continue to endanger our cognitive autonomy and unhampered opinion formation^6^. Diurnal variations in one’s reaction are not commonly discussed and may be abused by those purposefully spreading mis- and disinformation, be it explicitly or as a latent factor. A deeper scientific understanding of user response to potentially disinformative content can, however, also aid in the prevention of an unwitting contribution to such campaigns.

Specifically, we extrapolate two main takeaways from our study: Firstly, user activity on social media throughout the day can be mapped to pseudo-chronotypes on the morningness-eveningness continuum. We find these activity patterns to be a predictor of one’s propensity to spread potentially disinformative content and the constituent content types. *Evening types* have the highest inclination towards spreading potentially disinformative content, *infrequent posters* the lowest. Secondly, the spread of potentially disinformative content is negatively correlated with diurnal activity.

Generally, our findings are in line with previous literature detailing the link between cyclical behavioural patterns and Twitter use^41,59–61^ as well as with findings associating sunlight with cognitive function (and by extension critical thinking)^46^ and with activity on Twitter^45,47^. Similar patterns of diurnal activity archetypes have been identified in other studies. Piccardi et al.^75^, using principal component analysis (PCA) Wikipedia consumption patterns, found four principal components akin to our four behavioral clusters. Their PC2 had the largest weight in the morning (similar to our *morning type*), and PC4 had the largest weight in the evening (similar to our *evening type*). They also found one principal component with bimodal peaks (PC3, our *intermediate type)* and one with relatively flat behavior (PC1, our *infrequent posters*). The same study analyzed typical access times for topics. Some topic peak average times fall the identified interval of 3:15 am and 4:15 am, where ratios of potentially disinformative content peaks for *intermediate* and *evening types* as well as for *infrequent posters*. These topics include space, software, internet and culture, military and war, and society (see their Figure 9)^75^. Around 9:45 pm, the peak times of potentially disinformative content of *morning types*, topics are more media-centric including television, radio and literature^75^.

These results have implications for (**a**) our understanding of user responses to potentially disinformative information in relation to user activity and time of day, and (**b**) the design of interventions to prevent the spread of mis- and disinformation on social media.

There are two main theoretical explanations for susceptibility to mis- and disinformation. The first is the “inattention account”, which argues that people aim to share accurate content but are distracted from accuracy-focused decisions by the context of social media. The inattention account draws from dual-process theories of cognition. In contrast, the “motivated cognition” or “identity-protective account” posits that people consider not just accuracy of new information but also the goals served by accepting it as true. Both accounts face significant critiques and limitations, such as failed replication of supportive results. For an in-depth review, we refer to^76^. Some evidence for the inattention account points to cognitive functions that might show circadian variation^77,78^. Motivated cognition, on the other hand, may be shaped by political identities or underlying values. Some studies have linked political ideology to diurnal variations^79,80^, and sleep loss to reduced altruistic behavior^58^. Some cognitive control processes which may be involved in the ability to override pre-existing identities or values when evaluating new information fluctuate across the day. For example, self-monitoring of executive functions shows circadian variations^81^. Overall, both theories are based on thinking processes that are subject to diurnal variation. The evidence is more ample and robust for analytical thinking than for motivated reasoning, though. Our findings on the spread of misinformation being subject to diurnal variation can therefore be interpreted through the lens of either theory.

We found that potentially disinformative content is most likely to be spread around inferred bedtime, at 9:45 pm for *morning type* users and between 3:15 and 4:15 am for other users. This falls towards the end and after inferred waking time for all clusters. This variation is inline with the inattention account, assuming that *morning type* users would deplete their cognitive resources earlier in the night. The overall higher ratios of potentially disinformative content in *evening type* users can be contrasted with previous findings of reduced positive affect and social jetlag^82^. The peaks in the early morning may also stem from the fact that professional news outlets are usually not active during this time, reducing the portion of reliable content. Further research is needed to investigate the causes of the high share of potentially disinformative content during these times.

Our research may inform the timing of interventions against mis- and disinformation, and concentrate efforts on limited time frames. Continuously deploying interventions may be more costly for the implementer and may overload the user’s attentional capacity and patience. Shorter exposition may be more resource-effective and less intrusive. As a concrete example, social media companies could time interventions such as increasing communication friction (making it harder to react to posts without due thought^83^) or even throttling posting rates during those time ranges where users are particularly likely to spread misinformation (around 9:45 am for *morning types* and between 3:15 and 4:15 for other clusters). Similarly, the peak activity times of those users could be used to time preactive (inoculation, targeting the source of disinformation, and spreading truthful information in areas at risk of disinformation campaigns) or proactive (equipping members of the public with the skills to critically analyze and identify new information) interventions^6^ for greater reach in particular to those users most susceptible to potentially disinformative content (such as around 10:15 pm to target individuals with an evening preference). The potential of our findings to inform the design of protective measures is all the more relevant in light of the rising trend in cyber operations and information warfare^6,84^.

More specifically, in the context of COVID-19, the non-pharmaceutical interventions imposed by many countries, such as lockdowns, curfews and home office, have disrupted many peoples’ daily rhythms, plausibly giving rise to interaction effects between circadian mismatch and the course of the pandemic^85^ as well as aiding the spread of conspiracy theories^22,38^. Although potentially disinformative content posted per day and user increased for all clusters from the period outside of the lockdown to that within, the ratio of potentially disinformative content decreased. This can likely be attributed to a rise in reliable content due to increased informational coverage by mainstream and state media as well as by scientific research. Therefore, although we do not find evidence supporting that non-pharmaceutical interventions were followed by the increase in one’s propensity to spread mis- and disinformation, we cannot reject the possibility. We therefore continue to advice that future policy interventions consider their possible impact on human circadian activity to limit the risk of concomitant increases in mis- and disinformation^71^.

While a social media study allows the analysis of social dynamics at an unprecedented scale, it also comes with a set of limitations. In particular, using a dataset collected entirely from Twitter biases the reference population towards being more highly educated, working age, and male. The dataset, alongside its limitations, is discussed in detail in Gallotti et al.^71^. Our study is restricted to the context of Italy. Although we cross-reference with tweets originating from Germany (Supplementary Note A), our findings cannot be generalized further.

In terms of analysis, we use a set of proxy metrics: the ratio of potentially disinformative content (as a proxy for susceptibility to mis- and disinformation), activity patterns on Twitter (as a proxy for the user’s diurnal behavioural archetype), and average times of sunset and sunrise (as a proxy for sunlight exposure). These are computationally viable options allowing the large-scale analysis of behavioural phenomena but cannot measure the phenomena directly. However, social media data have a limited capacity to examine the underlying cognitive processes related to information spreading. Controlled behavioural experiments would allow a more direct measure of underlying cognitive processes.

Similarly, causality is yet to be established for the impact of time of day, diurnal tweeting behaviour, and non-pharmaceutical interventions against COVID-19 on one’s susceptibility to mis- and disinformation. Further challenges include an extension and comparison across countries, languages, platforms, and representative user groups. On a larger scale, we hope for further research into how knowledge of the diurnal patterns of our reaction to mis- and disinformation can effectively be leveraged and integrated into the design of interventions against large-scale manipulation. Temporality, along with other factors impacting our susceptibility to mis- and disinformation, is likely already modeled in the latent space of deep learning systems. An analytic understanding can aid us in maintaining integrity of mind and autonomy of thought.

## Methods

### Data

We consider a Twitter dataset^71^ collected through the Twitter Filter API based on a set of hashtags and keywords surrounding the Covid-19 pandemic, specifically *coronavirus, ncov, #Wuhan, covid19, covid-19, sarscov2, covid*. Analysis was limited to the time span of January 22, 2020, when more than 6000 cases were reported in China, up to August 1st 2022. Twitter restrictions limit collection to no more than 4.5 million messages per day, on average. 9128 tweets collected between January and February 2021 were not associated with a tweet type on collection and were excluded from analysis. After removal of duplicates and posts by users identified as bots, our body of analysis encompassed 18,148,913 tweets, retweets or replies, of which 1,001,045 are assigned a known reliability.

### Source reliability mapping

Tweets were assigned a source reliability rating by the dataset authors^71^ based on web domains, manually classified by experts, listed in multiple public databases, including journalistic and scientific sources^86–94^. From these sources, the authors created a database of 3892 domains after cleaning and processing. These different sources have been aligned by Gallotti et al.^71^ to a common classification scheme based on a *Harm Score* (HS), an ordinal classification of sources in terms of their potential contribution to manipulative and misinformative information spreading. Generally, a high Harm Sore indicated a more systematic and intentionally harmful knowledge manipulation and data fabrication. The news media web domains listed were divided into nine different categories of increasing Harm Score:

1. Scientific,
2. Mainstream Media,
3. Satire,
4. Clickbait,
5. Other,
6. Shadow,
7. Political,
8. Fake and Hoax,
9. Conspiracy and Junk Science.

The categories of Shadow and Other were merged in this paper. Tweets containing a link are compared to domains in the database and classified according to domain reliability. The categories were adapted to fit the project focus and are detailed in Supplementary Table S1. In this work, we identify as potentially disinformative content messages sharing web domains with Harm Score $\ge 7$.

### Geographic and time zone mapping

Geocoding and geodata cleaning was conducted by the dataset authors^71^ based on the user’s self-declared location field *ArcGIS API*. Mapping errors (based, for example, on non-toponymous entries or website URLs) entries were removed by isolating single locations associated with many different unique location strings and data restricted to country-based granularity. Within this study, we use exclusively the data found to originate from Italy. By extension, we ported the time zone of content returned by the Twitter API to Central European Summer or Winter Time, respectively.

For the calculation of sunrise and sunset time, we relied on the latitude and longitude of location strings. For users who only listed “Italy” as their location, the coordinates are approximated around the geographical centre of the peninsula. To preserve user anonymity, these strings were mapped the centroids of the 2021 territorial units of level 3 released by Eurostat^73^, defining provinces and metropolitan cities. For locations outside of level 3 provinces in Italy, we used the centroid of the closest territory. For locations equidistant from multiple territories, we chose the midpoint of these centroids.

### Clustering

Let $T=\{[t, t+\frac{1}{4})\ |\ 4t \in {\mathbb {N}} \wedge \ 0 \le t < 24 \}$ be the set of 15 minute intervals within a day given in hours, *F* the set of content types and *I* the set of users authoring content. We will subsequently use *t* to refer to one such interval $[t, t+\frac{1}{4}) \in T$ for simplicity. Let then $\{ P_{(t,i,f)} \}_{(t, i, f) \in T \times I \times F}$ be the set of posts of content type $f \in F$ authored during interval $t \in T$ by user $i \in I$, indexed by a surjective function from $T \times I \times F$ onto *P*.

We define a user’s activity level during a time interval $t \in T$ as the proportion of posts authored during this time interval as compared to the sum of posts authored overall.

1 $$\begin{aligned} a(t,i) = \frac{\sum \limits _{f \in F} \vert {P_{(i,t,f)}}\vert }{\sum \limits _{s \in T, \ f \in F}\vert {P_{(s,i,f)}}\vert } \end{aligned}$$

The activity levels were smoothed using a rolling average over a 90 minute Gaussian window with $\sigma =3$ (looping the values around midnight) and used for subsequent clustering. Six cluster performance indicators (specifically, Context-Independent Optimality^95^, Caliñski-Harabasz^96^, Davies-Boulin^97^, generalised Dunn^98^ and Silhouette^99^) informed our choice of cluster method and number of clusters. The indicators showed highest scores for k-means clustering with 3 distinct clusters with unique patterns of posting activity (*morning, intermediate* and *evening type* posters). Users with low post rates ($<240$ posts) are separated into a fourth cluster (*infrequent type posters*). We receive similar clusters when considering only posts by unverified users (Supplementary Note B).

Inter- and intra-cluster distances are detailed in Supplementary Table S4a, general information about the clusters is given in Supplementary Table S4b.

### Diurnal cluster activity

Let *C* be the set of all clusters where $c \in C$ is a subset of *I*. Function

2 $$\begin{aligned} a_c(t) =\frac{1}{\vert {c}\vert } \sum _{i \in c} a(t,i) \end{aligned}$$

calculates the activity levels during an interval *t* by cluster *c* where each user’s activity level carries the same weight. To denoise and compare the cluster activity curves, we transform them from the time domain into the frequency domain using the discrete Fourier transform:

3 $$\begin{aligned} X^c_k = \sum ^{N-1}_{n=0} a_{c,n} e^{-\frac{i 2 \pi }{N} kn} \qquad k \in [0, N-1] \end{aligned}$$

where $a_{c,n}=a_c(t_k)$ and $t_k = k\Delta$. Equation 3 yields a sequence of complex numbers $\{X_k^c\} = X^c_0, X^c_1,\ldots , X^c_{N-1}$ which describe amplitude and phase of sinusoidal functions. On summation, the sequence produces the original discrete signal. In particular, the $k^{th}$ Fourier coefficient provides information about the sinusoid that has *k* cycles over the given number of samples.

We then identified the coefficients with the greatest amplitude. Let $\{A^c \} = \{A^c_1, A^c_2,\ldots , A^c_{N-1}\}$ be the set of all amplitudes of the constituent sinusoidal functions for frequencies $0, 1,\ldots , N$, and let $\{ A^{(c,m)} \} \subset \{A^c \}$ be the set of *m* largest amplitudes.

The signal is then recombined as follows to contain only the harmonics with *m* greatest amplitudes:

4 $$\begin{aligned} \ h^c(n, t)&=A^c_n \cos {\frac{2\pi }{P^c}nt - \varphi _n^c} \end{aligned}$$

5 $$\begin{aligned} S^{c,m}_N(t)&\approx \frac{A^c_0}{2} + \sum ^{N}_{n=1} \left\{ \begin{aligned}&h^c(n, t){} & {} \text {if} \quad A^c_n \in \{ A^{(c,m)} \} \\&0{} & {} \text {otherwise} \end{aligned} \right. \end{aligned}$$

where *h*(*n*, *t*) describes the $n^{th}$ harmonic of the Fourier series. $P^c$ is the period of function *a*(*t*, *c*), $A^c_n$, $\varphi ^c_n$ and $\frac{n}{P^c}$ are amplitude, phase and frequency of harmonic $h^c(n, t)$ respectively, and $S^{c,m}_N(t)$ approximates the recomposed signal at time point *t*.

We used the value for *m* where the change in distance to the next larger value grew smaller for each cluster. If two values are supported by an equal number of indicators, we chose the smaller one. Let $\{ U \}$ be a set of 7 distance metrics, specifically Partial Curve Mapping^100^, the area method^101^, discrete Frechet distance^102^, curve length^103^, Dynamic Time Warping^104^ as well as mean absolute error and mean squared error. Let then $\{ D_{u}^m \} = { \sum _{t \in T} u(S^{(c,m)}_N(t), a^c(t)) }$ describe the distances between the original signal and the reconstruction (see Equation 1 and Equation 5, respectively) for a given value of *m* and a distance metric $u \in U$. For a cluster *c*, we find the value of *m* as:

6 $$\begin{aligned} m^c = \min \{ \text {mode} \{ \underset{m \in [1,4]}{arg min} \ (D_u^{(c,m)}, D_u^{(c,m-1)} ) \} \} \end{aligned}$$

where $\underset{m \in M}{arg min}\ \ h(m) = \{ m \ | \ h(x) \ge h(m) \ \forall x \in M \}$ returns the set of points *m* for which a function *h*(*m*) returns the function’s smallest value, if it exists. The *mode* operation returns the set of most common elements, and *min* finds the minimum element of a set. We accordingly used $m=3$ for all clusters.

Recomposing the signal $a_c(t)$ (Eq. 2) in this manner leaves us with the set of smoothed diurnal cluster activity values $\{ S_{(t,c)} \}_{(t,c) \in T x C}$.

Details on the maxima and minima are found in Supplementary Table S2.

*Potentially disinformative cluster activity* as shown in Supplementary Fig S1 is calculated following the same process as described for overall diurnal activity, restricting the considered content types to potentially disinformative ones.

Let $F^H$ denote the set of potentially disinformative content types, consisting of conspiracy or junk science, fake or hoax news, and politically biased news.

Then, $a_c^H(t)$ is then:

7 $$\begin{aligned} a^H(t,i)&= \frac{\sum \limits _{f \in F^H} \vert {P_{(i,t,f)}}\vert }{\sum \limits _{s \in T, \ f \in F^H}\vert {P_{(s,i,f)}}\vert } \end{aligned}$$

8 $$\begin{aligned} a_c^H(t)&= \frac{1}{\vert {c}\vert } \sum _{i \in c} a^H(t,i) \end{aligned}$$

To find the smoothed set of potentially disinformative diurnal cluster activity $\{ S^H_{(t,c)} \}_{(t,c) \in T x C}$, a rolling average over a 90 minute Gaussian window ($\sigma =6$) was applied to this signal, looping the values around midnight. The process described in Eqs. (3)–(6) is then applied to the activity levels defined in Eq. (7) with $m=4$ for *intermediate type* users and $m=3$ for all other clusters, resulting in the set of smoothed potentially disinformative diurnal cluster activity $\{ S^H_{(t,c)} \}_{(t,c) \in T x C}$.

### Periods of heightened activity and prolonged wakefulness

To find the periods of heightened activity, let

9 $$\begin{aligned} i(t,n) = (t+n)(\bmod \ 24) \end{aligned}$$

return the time of day *n* hours past *t* where $\bmod$ refers to the modulo operator. Then, let

10 $$\begin{aligned} j(t,s,n) = {\left\{ \begin{array}{ll} t< s \wedge s< i(t,n) &{} \text {if } t< i(t,n)\\ s > t \vee s < i(t,n) &{} \text {otherwise} \end{array}\right. } \end{aligned}$$

indicate whether a time point *s* occurs within *n* hours past *t*. Then, the onset of heightened activity for cluster *c* and for $n=16$ is found by:

11 $$\begin{aligned} g(c, n) = \underset{t \in T}{arg max}\ \ \sum _{s \in T \wedge j(s,t,n)} A_{(s,c)} \end{aligned}$$

Analogously to the *argmin* operation, the set of points *t* for which a function *h*(*t*) returns the function’s largest value, if it exists, is found as:

12 $$\begin{aligned} \underset{t \in T}{arg max}\ \ h(t) = \{ t \ | \ h(x) \le h(t) \ \forall x \in T \} \end{aligned}$$

The end of the period of heightened activity is then *i*(*g*(*c*, *n*), *n*). Supplementary Table S3 lists these times for each cluster. We refer to the period after the end but before the onset of heightened activity as *prolonged wakefulness*.

### Content type ratios

We calculate the ratio of a given content type without including the category “Other”, which is not easily classifiable, makes up the vast majority of content in our dataset, and could possibly obstruct patterns in the data.

Let $F^K$ be the subset of *F* without “Other”. The ratio for content type $f \in F^K$, cluster *c* and 15 minute time interval within a day *t* is the average user ratio of that content type within a cluster:

13 $$\begin{aligned} r(t,i,f)&= \frac{\vert {P_{(t,i,f)}}\vert }{\sum _{g \in F^K}\vert {P(t,i,g)}\vert } \end{aligned}$$

14 $$\begin{aligned} r(t,c,f)&= \frac{1}{\vert {c}\vert } \sum _{i \in c}r(t,i,f) \end{aligned}$$

The ratio of potentially disinformative content is then:

15 $$\begin{aligned} r^H(t,i)&= \frac{\sum _{f \in F^H}\vert {P_{(t,i,f)}}\vert }{\sum _{f \in F^K}\vert {P(t,i,f)}\vert } \end{aligned}$$

16 $$\begin{aligned} r^H(t, c)&= \frac{1}{\vert {c}\vert } \sum _{i \in c}r^H(t,i) \end{aligned}$$

where $F^H$ is again the set of potentially disinformative content types, consisting of conspiracy or junk science, fake or hoax news, and politically biased news, and is a subset of $F^K$.

We applied the process described by Eqs. (1)–(6) also to the diurnal pattern of ratios of potentially disinformative content. Given the noisy nature of the ratio curves, we applied a round of rolling Gaussian smoothing ($\text {window}=6, \sigma =3$) to the curves $r^H(t,i)$ before further processing. On these curves, the values of *m* for Eq. (5) preceding the lowest change in distance metrics were $m=4$ for *intermediate type* users, and $m=3$ for all other types. We refer to the set of smoothed diurnal ratios of potentially disinformative content as $\{ R_{(t,c)} \}_{(t,c) \in T x C}$. We consider a time span *t* to reflect an increased susceptibility to spreading potentially disinformative content for a given cluster if the smoothed ratio $R_{(t,c)}$ is greater than the third quartile. So *t* is a time of increased susceptibility for cluster *c* if $Pr[\{ R_{(s,c)} | s \in T \} < R_{(t,c)}] \le 3/4$, where *Pr* refers to the probability of an occurrence.

### Statistics

$\chi ^2$-test was used for comparison of nominal variables, i.e. the relationship in between times of lockdown and potentially disinformative content and in between content type and cluster affiliation. We used the Dip Test of Unimodality^105^ to test unimodality of distributions of diurnal activity for each cluster. Unimodality could be rejected for all clusters both for the smoothed diurnal activity curves of set $\{ A_{(t,c)} \}_{(t,c) \in T x C}$ and for the raw activity aggregations over the day described by Eq. (2). See Supplementary Table S3 for the Dip statistic and $p$-values per cluster.

While we assume a monotonic relationship between the number of posts per user and the ratio of potentially disinformative content, we do not assume a linear one. Therefore, we use Spearman’s $\rho$ to describe correlation between these variables (Table 2a). The same is true for correlation of user activity throughout the day with ratio of potentially disinformative content throughout the day. Table 2b shows the correlation coefficient and $p$-value for the raw activity aggregations over the day and for the smoothed activity curves.

Neither diurnal activity nor diurnal ratio of potentially disinformative content types are normally distributed (Shapiro-Wilk $W=0.875$, $p$-value$>0.001$ and $W=0.886$, $p$-value$>0.001$, respectively). Therefore, we used the nonparametric Mann-Whitney *U* test to assess the difference in distributions of ratios of potentially disinformative content throughout the day by cluster (Table 1) and between day and nighttimes (table 3).

### Supplementary Information

Supplementary Information.

## Data Availability

This paper uses data generated by Gallotti et al. ^71^ available from the second author on reasonable request. The derived aggregated and anonymized data as well as the analysis supporting the findings of this study are openly available at: https://github.com/ethz-coss/diurnal-misinformation.
