# Supplementary material for: Early morning hour and evening usage habits increase misinformation-spread
Source: Sci Rep. 2024 Aug 30;14:20233. doi: 10.1038/s41598-024-69447-8 (PMC11364767; doi:10.1038/s41598-024-69447-8)
Supplement: Supplementary file 1 — Supplementary Information. [file 41598_2024_69447_MOESM1_ESM.pdf]

Supplementary Information for: Early morning hour and evening usage habits increase misinformation-spread

Elisabeth Stockinger[1,*], Riccardo Gallotti[2], and Carina I. Hausladen[1]

[1]Computational Social Science, Department of Humanities, Social and Political Sciences, ETH Zurich, Zurich, 8092, Switzerland
[2]Complex Human Behaviour Lab, Fondazione Bruno Kessler, Trento, 38123, Italy
[*]elisabeth.stockinger@live.at

Supplementary Fig. S1. Smoothed diurnal activity of potentially disinformative content per cluster. For each cluster, the two highest peaks of activity are stressed and annotated with the time of occurrence.

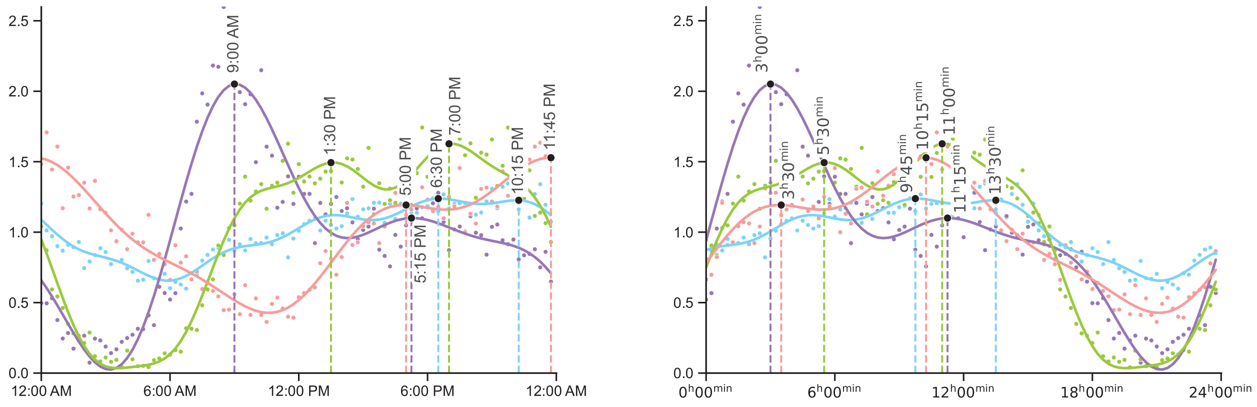

(a) potentially disinformative activity by time of day **(b)** potentially disinformative activity by inferred waking time

Supplementary Fig. S2. Number of potentially disinformative and likely not disinformative posts per cluster (first row) as well as the coarse average ratio of potentially disinformative content per user in each cluster (second row, Equation 15). The content ratios were fitted with a regression line of order 5. The shaded area around this line is the 95% confidence interval.

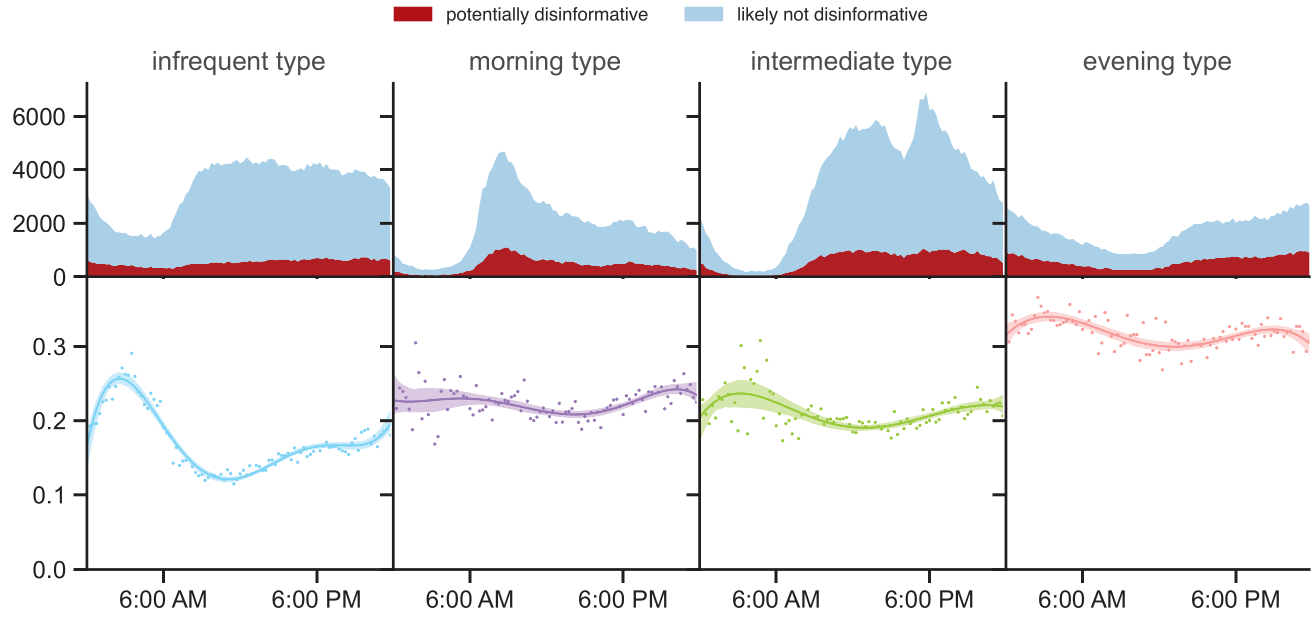

Supplementary Table S1. Classification of social media content adapted from Gallotti *et al.* (2020)[1]. The term "potentially disinformative" refers to those categories of concern to democratic opinion formation. Each content category is listed alongside general statistics and ratio of posts per cluster. Ratios are averaged over the individual users as defined in Equation 13. Ratios of potentially disinformative content are notably elevated for *evening types* compared to all other clusters.

Category	Characteristics	total posts	mean posts per author	median posts per author	ratio			
					infrequent	morning	intermediate	evening
Science	subject to a rigorous validation process by scientific methods	18,831	2,261	484	0.023	0.020	0.020	0.022
Mainstream media	subject to fact checking and media accountability	757,661	2,682	672	0.761	0.763	0.786	0.669
Satire	distorts or misrepresents information for entertainment value, usually is easily identified	4,301	734	170	0.008	0.005	0.007	0.004
Clickbait	generally distorts or intentionally misrepresents information to capture attention	12,197	735	39	0.041	0.008	0.006	0.009
Other	general-purpose category collecting content which is not easily classifiable, includes links that are anonymised and often temporary for higher obscurity (originally "Shadow"), or does not contain links at all	17,210,987	1,787	368	-	-	-	-
Politically biased	aims to build a consensus on a polarised position by omission, manipulation or distortion of information	98,700	2,755	721	0.122	0.080	0.069	0.174
Fake or hoax	entirely fabricated or manipulated content that aims to be perceived as realistic and reliable	43,888	2,601	1,143	0.027	0.045	0.041	0.061
Conspiracy & junk science	strongly ideological, inflammatory content alternative or oppositional to tested and accountable knowledge and information with the intent of building echo chambers	65,661	4,275	1,679	0.019	0.078	0.072	0.062
Potentially disinformative	*composite category of politically biased information, fake or hoax news, and conspiracy and junk science*	*208,249*	*3,202*	*1,110*	*0.167*	*0.203*	*0.182*	*0.297*

Supplementary Table S2. Times of maximum and minimum of activity, both overall and potentially disinformative, as well as of ratios of potentially disinformative content per cluster sorted by extremity, i.e. the first row per cluster shows the largest maximum and smallest minimum.

		max			min		
		clock time	time past awakening	activity/ratio	clock time	time past awakening	activity/ratio
activity	infrequent	19:00	10h 15min	0.013	5:45	21h 00min	0.007
		14:00	5h 15min	0.012	16:00	7h 15min	0.012
	morning	9:30	3h 30min	0.022	3:15	21h 15min	<0.001
		17:00	11h 00min	0.012	14:45	8h 45min	0.011
	intermediate	18:45	10h 45min	0.017	4:30	20h 30min	<0.001
		12:30	4h 30min	0.017	15:30	7h 30min	0.016
	evening	23:15	9h 45min	0.016	10:15	20h 45min	0.004
		16:45	3h 15min	0.012	17:45	4h 15min	0.012
potentially disinformative content ratio	infrequent	3:15	18h 30min	0.253	10:00	1h 15min	0.121
		19:45	11h 00min	0.168	21:00	12h 15min	0.167
	morning	21:45	15h 45min	0.244	15:30	9h 30min	0.202
		1:30	19h 30min	0.235	12:00	6h 00min	0.208
		5:45	23h 45min	0.232	3:45	21h 45min	0.220
		9:30	3h 30min	0.230	7:45	1h 45min	0.222
		18:15	12h 15min	0.227	19:15	13h 15min	0.225
		13:30	7h 30min	0.212	-	-	-
	intermediate	4:15	20h 15min	0.249	16:15	8h 15min	0.188
		23:00	15h 00min	0.227	13:15	5h 15min	0.193
		10:00	2h 00min	0.212	7:30	23h 30min	0.194
		14:00	6h 00min	0.193	1:15	17h 15min	0.215
	evening	3:45	14h 15min	0.341	14:15	0h 45min	0.297
		19:30	6h 00min	0.322	22:45	9h 15min	0.315
potentially disinformative activity	infrequent	18:30	9h 45min	0.013	6:00	21h 15min	0.007
		22:15	13h 30min	0.013	15:30	6h 45min	0.011
		13:45	5h 00min	0.012	20:30	11h 45min	0.012
	morning	9:00	3h 00min	0.021	3:15	21h 15min	<0.001
		17:15	11h 15min	0.011	14:15	8h 15min	0.010
	intermediate	19:00	11h 00min	0.017	3:30	19h 30min	<0.001
		13:30	5h 30min	0.016	16:00	8h 00min	0.014
	evening	23:45	10h 15min	0.016	10:30	21h 00min	0.004
		17:00	3h 30min	0.012	18:45	5h 15min	0.012

Supplementary Table S3. Dip-test of modality of user activity curves (panels *coarse* and *smooth*) as well as times of onset and end of heightened activity per cluster. Significant results (p-value < 0.05) are given in bold font.

	coarse		smooth		heightened activity	
	dip statistic	p-value	dip statistic	p-value	onset	end
infrequent	0.059	**0.014**	0.054	**0.035**	8:45 AM	12:45 AM
morning	0.058	**0.015**	0.028	0.890	6:00 AM	10:00 PM
intermediate	0.079	**>0.001**	0.055	**0.030**	8:00 AM	12:00 AM
evening	0.053	**0.042**	0.035	0.566	1:30 PM	5:30 AM

Supplementary Table S4. Cluster statistics

(a) Distance metrics for the ratio of potentially disinformative content when aligned by features of the cluster activity curves. The minimum feature for each metric is indicated in bold font. See[2–6] for details on the individual metrics.

	Partial Curve Mapping	discrete Frechet distance	area between curves	curve length	Dynamic Time Warping	mean absolute error	mean squared error
clock time	4.08e+01	1.30e-01	1.95e+00	4.02e+00	7.85e+00	4.09e-02	**4.72e-03**
min activity	2.61e+01	**1.24e-01**	1.96e+00	**3.87e+00**	7.88e+00	4.10e-02	4.81e-03
max activity	3.63e+01	1.27e-01	1.97e+00	3.95e+00	7.96e+00	4.15e-02	4.90e-03
first inflection	1.72e+01	1.26e-01	**1.92e+00**	4.00e+00	**7.78e+00**	**4.05e-02**	4.83e-03
first peak	2.84e+01	1.26e-01	1.94e+00	3.89e+00	7.86e+00	4.09e-02	4.76e-03
steepest ascent	1.72e+01	1.26e-01	**1.92e+00**	4.00e+00	**7.78e+00**	**4.05e-02**	4.83e-03
increased activity	**1.65e+01**	1.25e-01	1.93e+00	4.12e+00	7.85e+00	4.09e-02	4.81e-03

(b) Statistics on posts, users, and posts per user for each cluster as well as distances between and within cluster activity curves. The maximum distance within a cluster is indicated in bold font.

	users	posts	posts per user	distances			
				infrequent	morning	intermediate	evening
infrequent	860,228	7,858,209	9.14	**0.408**	-	-	-
morning	3,155	2,815,887	892.52	0.058	**0.131**	-	-
intermediate	5,461	5,199,655	952.14	0.044	0.061	**0.111**	-
evening	2,654	2,338,475	881.11	0.035	0.090	0.073	**0.158**

Supplementary Notes

Supplementary Note A User Activity Clustering in Germany

To ensure robustness of clustering method and conclusions, we cross-analysed user activity on Twitter originating within Germany. Our dataset encompassed 18,162,387 Tweets, Retweets and Replies authored within the same time span as our main corpus of Tweets originating from Italy (January 22nd, 2020, up to August 1st, 2022).

As in Italy, the majority of cluster performance indicators suggested k-means clustering into 3 clusters, resulting in qualitatively similar results (Supplementary Fig. S3, Supplementary Table S5a). Following the same naming convention, the waking times differ from those of their Italian counterparts by an hour at most (Supplementary Table S5b). The ratios of potentially disinformative content differ more strongly in between Germany and Italy. Ratios of potentially disinformative content are generally lower than they are in Italy. The peak of potentially disinformative content spreading of *morning type* users falls shortly after midnight as opposed to 9:45 pm in Italy (Supplementary Table S5c).

Supplementary Note B Behaviour of verified and unverified users

Verified and unverified users exhibit some structural differences in their posting habits. The distributions of potentially disinformative posts are significantly different ($\chi^2 = 8801.25$, p-value$< .001$), with verified users posting more reliable content (Supplementary Table S6).

Clustering only unverified users results in extremely similar clusters to those found when clustering independently of verification status (Supplementary Fig. S4).

Supplementary Fig. S3. Smoothed diurnal activity aligned by waking time, and ratio of potentially disinformative content aligned by clock time across clusters in Germany (solid) and Italy (dotted). For each cluster, the one to two highest peaks of activity and the highest ratio are stressed and annotated with time of occurrence.

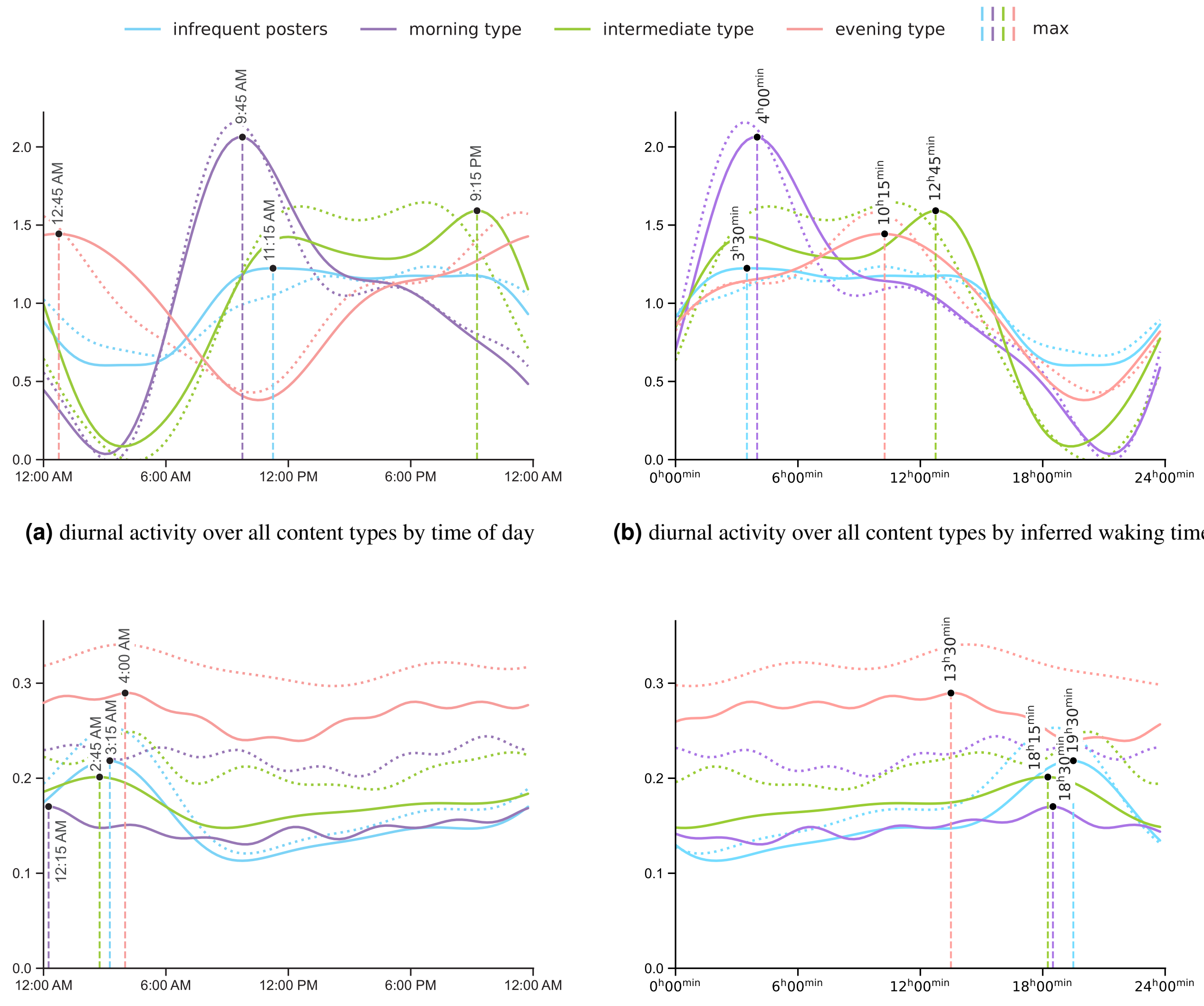

(a) diurnal activity over all content types by time of day

(b) diurnal activity over all content types by inferred waking time

(c) ratio of potentially disinformative content by time of day

(d) ratio of potentially disinformative content by inferred waking time

Supplementary Table S5. Statistics on clusters formed by Tweets originating from Germany.

(a) Statistics on posts, users, and posts per user for each cluster as well as distances between and within cluster activity curves. The maximum distance within a cluster is indicated in bold font.

	users	posts	posts per user	distances			
				infrequent	morning	intermediate	evening
infrequent	911,795	8,554,176	9.38	**0.410**	-	-	-
morning	4,143	3,320,162	801.39	0.047	**0.147**	-	-
intermediate	5,081	4,178,039	822.29	0.029	0.056	**0.136**	-
evening	2,315	2,110,010	911.45	0.050	0.093	0.068	**0.144**

(b) Dip-test of modality of user activity curves (panels *coarse* and *smooth*) as well as times of onset and end of heightened activity per cluster in Germany. Significant results (p-value$< .05$) are given in bold font.

	coarse		smooth		heightened activity	
	dip statistic	p-value	dip statistic	p-value	onset	end
infrequent	0.070	**0.001**	0.075	**0.000**	7:45 AM	11:45 PM
morning	0.060	**0.011**	0.030	0.832	5:45 AM	9:45 PM
intermediate	0.061	**0.009**	0.053	**0.043**	8:30 AM	12:30 AM
evening	0.049	0.093	0.037	0.438	2:30 PM	6:30 AM

(c) Times of maximum and minimum activity as well as of the two highest maximum and lowest minimum ratios per cluster in Germany.

		max			min		
		clock time	time past awakening	activity/ratio	clock time	time past awakening	activity/ratio
activity	infrequent	11:15	3h 30min	0.013	3:00	19h 15min	0.006
		21:00	13h 15min	0.012	16:00	8h 15min	0.012
	morning	9:45	4h 00min	0.021	3:00	21h 15min	<0.001
	intermediate	21:15	12h 45min	0.017	4:00	19h 30min	0.001
		12:00	3h 30min	0.015	16:45	8h 15min	0.013
	evening	0:45	10h 15min	0.015	10:30	20h 00min	0.004
potentially disinformative content ratio	infrequent	3:15	19h 30min	0.218	9:45	2h 00min	0.113
		19:15	11h 30min	0.148	20:45	13h 00min	0.147
	morning	0:15	18h 30min	0.170	10:00	4h 15min	0.130
		20:30	14h 45min	0.156	14:30	8h 45min	0.136
	intermediate	2:45	18h 15min	0.201	9:00	0h 30min	0.148
		19:30	11h 00min	0.174	21:00	12h 30min	0.173
	evening	4:00	13h 30min	0.290	12:30	22h 00min	0.239
		1:15	10h 45min	0.287	10:00	19h 30min	0.240
potentially disinformative activity	infrequent	22:00	14h 15min	0.013	5:30	21h 45min	0.007
		18:45	11h 00min	0.012	15:45	8h 00min	0.011
	morning	9:15	3h 30min	0.020	3:00	21h 15min	0.001
		17:00	11h 15min	0.012	14:30	8h 45min	0.012
	intermediate	21:45	13h 15min	0.017	4:00	19h 30min	0.001
		11:45	3h 15min	0.013	14:00	5h 30min	0.013
	evening	1:15	10h 45min	0.016	10:30	20h 00min	0.004
		2:00	11h 30min	0.016	9:45	19h 15min	0.004

Supplementary Fig. S4. Smoothed diurnal activity as well as the ratio of potentially disinformative content posted per cluster according to clusters formed only of unverified users. For each cluster, the one (or two) highest peaks of activity and ratio are annotated with their time of occurrence.

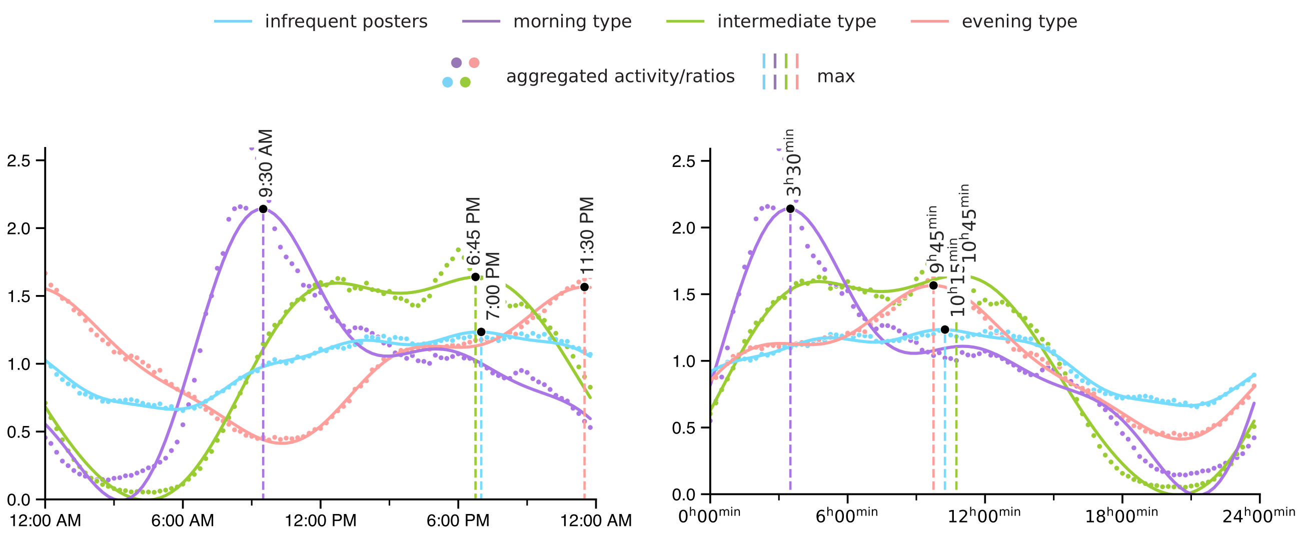

(a) diurnal activity over all content types by time of day
(b) diurnal activity over all content types by inferred waking time

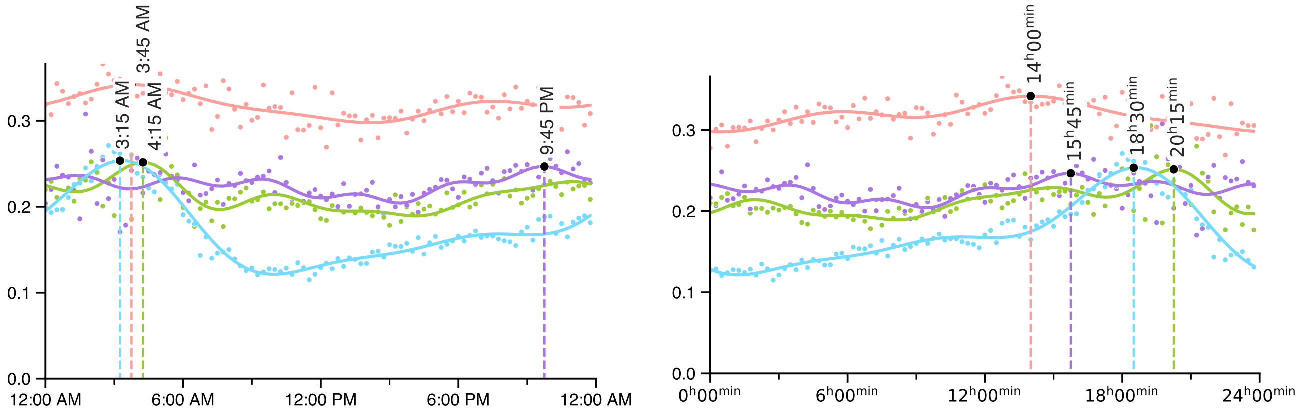

(c) ratio of potentially disinformative content by time of day
(d) ratio of potentially disinformative content by inferred waking time

Supplementary Table S6. Ratios of posts by content type and verification of user.

	ratio by Tweet		ratio by user	
	unverified	verified	unverified	verified
Science	0.019	0.006	0.022	0.032
Mainstream Media	0.748	0.974	0.759	0.872
Satire	0.004	< 0.001	0.008	0.001
Clickbait	0.013	< 0.001	0.039	0.001
Politically biased	0.102	0.013	0.120	0.076
Fake or hoax	0.045	0.004	0.028	0.011
Conspiracy & junk science	0.068	0.003	0.023	0.007
Potentially disinformative	*0.216*	*0.020*	*0.171*	*0.093*

References

1. Gallotti, R., Valle, F., Castaldo, N., Sacco, P. & De Domenico, M. Assessing the risks of 'infodemics' in response to COVID-19 epidemics. *Nat. Hum. Behav.* **4**, 1285–1293, DOI: 10.1038/s41562-020-00994-6 (2020).

2. Witowski, K. & Stander, N. Parameter identification of hysteretic models using Partial Curve Mapping. In *12th AIAA Aviation Technology, Integration and Operations (ATIO) Conference and 14th AIAA/ISSMO Multidisciplinary Analysis and Optimization Conference*, DOI: 10.2514/6.2012-5580 (2012).

3. Fréchet, M. M. Sur quelques points du calcul fonctionnel. *Rendiconti del Circolo Matematico di Palermo* **22**, 1–72, DOI: 10.1007/BF03018603 (1906).

4. Jekel, C. F., Venter, G., Venter, M. P., Stander, N. & Haftka, R. T. Similarity measures for identifying material parameters from hysteresis loops using inverse analysis. *Int. J. Material Form.* **12**, 355–378, DOI: 10.1007/S12289-018-1421-8/FIGURES/29 (2019).

5. Andrade-Campos, A., De-Carvalho, R. & Valente, R. A. F. Novel criteria for determination of material model parameters. *Int. J. Mech. Sci.* **54**, 294–305, DOI: 10.1016/J.IJMECSCI.2011.11.010 (2012).

6. Berndt, D. & Clifford, J. Using Dynamic Time Warping to Find Patterns in Time Series. *Proc. ACM SIGKDD Int. Conf. on Knowl. Discov. Data Min.* (1994).
